# Quantitative microbial risk assessment for waterborne pathogens in a wastewater treatment plant and its receiving surface water body

**DOI:** 10.1186/s12866-020-02036-7

**Published:** 2020-11-12

**Authors:** Joshua Mbanga, Akebe Luther King Abia, Daniel Gyamfi Amoako, Sabiha. Y. Essack

**Affiliations:** 1 Antimicrobial Research Unit, College of Health Sciences, University of Kwazulu-Natal, Private Bag X54001, Durban, 4000 South Africa; 2 Department of Applied Biology and Biochemistry, National University of Science and Technology, P.O Box AC 939 Ascot, Bulawayo, 00263 Zimbabwe

**Keywords:** Quantitative microbial risk assessment, Wastewater treatment plants, *Escherichia coli*, *Enterococcus*

## Abstract

**Background:**

Access to safe water for drinking and domestic activities remains a challenge in emerging economies like South Africa, forcing resource-limited communities to use microbiologically polluted river water for personal and household purposes, posing a public health risk. This study quantified bacterial contamination and the potential health hazards that wastewater treatment plant (WWTP) workers and communities may face after exposure to waterborne pathogenic bacteria in a WWTP and its associated surface water, respectively.

**Results:**

*Escherichia coli* (Colilert®-18/ Quanti-Tray® 2000) and enterococci (Enterolert®/ Quanti-Tray® 2000) were quantified and definitively identified by real-time polymerase chain reaction targeting the *uidA* and *tuf* genes, respectively. An approximate beta-Poisson dose-response model was used to estimate the probability of infection (Pi) with pathogenic *E. coli*. Mean *E. coli* concentration ranged from 2.60E+ 02/100 mL to 4.84E+ 06/100 mL; enterococci ranged from 2.60E+ 02/100 mL to 3.19E+ 06/100 mL across all sampled sites. Of the 580 *E. coli* isolates obtained from this study, 89.1% were intestinal, and 7.6% were extraintestinal pathogenic *E. coli*. The 579 enterococci obtained were 50.4% *E. faecalis* (50.4%), 31.4% *E. faecium*, 3.5%, *E. casseliflavus* and 0.7% *E. gallinarum*. The community health risk stemming from the use of the water for recreational and domestic purposes revealed a greater health risk (Pi) from the ingestion of 1 mL of river water from upstream (range, 55.1–92.9%) than downstream (range, 26.8–65.3%) sites. The occupational risk of infection with pathogenic *E. coli* for workers resulting from a once-off unintentional consumption of 1 mL of water was 0% (effluent) and 23.8% (raw influent). Multiple weekly exposures of 1 mL over a year could result in a Pi of 1.2 and 100% for the effluent and influent, respectively.

**Conclusion:**

Our findings reveal that there is a potentially high risk of infection for WWTP workers and communities that use river water upstream and downstream of the investigated WWTP.

**Supplementary Information:**

The online version contains supplementary material available at 10.1186/s12866-020-02036-7.

## Background

Water shortage remains among the most pressing challenges the world currently faces. Over 844 million people do not have access to safe drinking water, and approximately 159 million still drink unprocessed water from surface water sources, including streams and lakes [1]. Low- and middle- income countries (LMICs) and emerging economies like South Africa, with densely populated urban centers and informal housing, tend to have inappropriate wastewater management systems, and these are set to experience greater pressure as the urban population increases [2]. Such populations resort to nearby water bodies like rivers for human and household waste disposal, leading to extensive pollution of these resources.

Faecal contamination of water with pathogenic bacteria, viruses, and protozoa remains one of the main causes of waterborne diseases as these microbes can persist in polluted water for protracted periods and ultimately cause infections [3, 4]. Fecal contamination of water is mainly assessed using fecal indicator bacteria such as *Escherichia coli* and enterococci [5]. Assessing water quality is crucial as it allows for investigations into the causes of pollution and averts likely waterborne diseases, by providing evidence on the risks of human exposure related to different water uses [6, 7].

Due to the possible health threats linked to the occurrence of microbial pollutants in water resources, wastewater treatment plants (WWTP) were created as central units to reduce the pollutant loads to acceptable limits before the discharge of the resultant effluent into receiving water bodies. The detection of pathogenic microbes in WWTP effluents is, therefore, a cause of concern, especially for those involved in public health and water administration [8]. People living and working near WWTPs in urban settings are frequently exposed to a broad range of microbial pathogens [9, 10].

Diarrhoeagenic strains of *E. coli* (DEC) are among the most notable pathogens that have been isolated from wastewater effluents and at sites downstream of WWTPs [9, 10]. Pathogenic *E. coli* can either result in extraintestinal or intestinal infections. Extraintestinal pathogenic *E. coli* (ExPEC) includes uropathogenic *E. coli* (UPEC), avian pathogenic *E. coli* (APEC), and neonatal meningitis *E. coli* (NMEC) [11, 12]. Intestinal pathogenic *E. coli* (InPEC) or DEC is categorized into six groups: entero-hemorrhagic (EHEC), entero-pathogenic (EPEC), entero-aggregative (EAEC), enterotoxigenic (ETEC), entero-invasive (EIEC), and diffusely adherent (DAEC) [9, 13].

South Africa’s rural, peri-urban and informal settlements in urban areas house communities that remain heavily dependent on rivers and other water bodies for their everyday water requirements, some of which are polluted by treated and untreated WWTP discharges [10, 14]. In KwaZulu-Natal province in South Africa the uMsunduzi river is utilised for a variety of anthropogenic activities including domestic, industrial, agricultural and municipal activities [15]. The river flows through the provincial capital Pietermaritzburg (total population 900,113) and has a catchment size of 875 km^2^ and a tributary length of 115 km as it discharges into the uMgeni river [15]. Pietermaritzburg is characterized by numerous informal settlements next to surface water sources including the uMsunduzi river [14] which also receives runoff from a major WWTP in the city. Previous studies revealed that uMsunduzi River water (Pietermaritzburg) was contaminated with faecal matter and therefore unsafe for domestic, recreational and, agricultural purposes [14].

The likely exposure routes from polluted water sources include intentional and accidental ingestion, skin contact, and inhalation of droplets [16]. While the people in resource-poor communities may be exposed to all these routes, workers at WWTPs are more likely exposed to pathogens through inhalation of contaminated droplets [17]. This is particularly the case where proper personal protective equipment is not used or where personnel are not well trained. Exposure to bioaerosols generated in a WWTP can occur through numerous routes. Intake may be via the oral route from splashes, contaminated foodstuff, any hand-to-mouth contact, and contaminated personal protective equipment. Intake might also be via the respiratory system through inhalation of bioaerosols [17] or the skin such as by penetration through damaged or broken skin as a result of injuries. However, it is assumed that dermal contact and inhalation are less significant compared to exposure through ingestion [18].

For the river sites, probable exposure was considered to be through accidental or intentional ingestion of untreated river water by communities living upstream and downstream of the WWTP. Intentional ingestion might be due to direct consumption of the river water in places without access to a water supply. Accidental ingestion might occur due to the deliberate use of the polluted river water for household chores (washing clothes and cleaning dishes, among others) without treatment, and use of the river water for recreational activities like swimming or bathing [6, 19].

An in-depth understanding of the reservoirs of pathogens and their persistence in the environment is crucial in assessing the risks these microbes pose to the health of humans, animals, and the environment [20]. Quantitative microbial risk assessment (QMRA) is used to approximate the likelihood of health hazards resulting from human contact with pathogenic microbes in the environment [7, 16]. QMRA has been used to determine the disease burden of pathogens [2, 6] and to assess the community health risks due to contact with polluted water [2, 6, 10, 21, 22]. QMRA is an essential tool as safe water for drinking, and domestic activities remains a challenge that is often exacerbated by inefficient and insufficient sanitation infrastructure [23]. It provides a vital basis for the implementation of measures to protect both community members and WWTP workers as there is scanty information on the potential risks linked to the regular use of and exposure to uMsunduzi River water. This study investigated probable direct exposure to pathogenic *E. coli*, which was assumed to occur mainly through ingestion and inhalation. This study aimed to (i) assess the microbial quality of treated and untreated wastewater from a major WWTP and its associated surface water in KwaZulu-Natal, South Africa and, (ii) estimate the potential human health hazards linked with exposure to waterborne pathogenic bacteria within the WWTP (workers’ occupational exposure) and river water used by the communities.

## Results

### Mean concentrations of *E. coli* and *Enterococcus* spp.

All 60 water samples analyzed were positive for both *E. coli* and *Enterococcus* spp. The mean counts for both organisms are presented in Table 1. The arithmetic mean was used to compute the health risk due to pathogenic *E. coli*.

**Table 1:** Average *Enterococcus* spp. and *E. coli* counts (MPN/100 mL) per month and site of collection

MONTH	Mean MPN/100 ml			
	INFLUENT	EFFLUENT	UPSTREAM	DOWNSTREAM
***Enterococcus***	(*n* = 15)	(*n* = 15)	(*n* = 15)	(*n* = 15)
May (*n* = 8)	2.90E+ 05	4.30E+ 03	4.08E+ 03	2.33E+ 03
June (*n* = 8)	3.89E+ 05	4.70E+ 02	1.73E+ 04	2.10E+ 03
July (*n* = 12)	9.67E+ 05	1.03E+ 03	2.85E+ 04	6.16E+ 03
August (*n* = 8)	1.75E+ 06	3.33E+ 03	1.99E+ 04	1.59E+ 03
September (*n* = 8)	3.19E+ 06	2.62E+ 03	2.04E+ 04	3.87E+ 03
October (*n* = 8)	2.53E+ 06	2.60E+ 02	4.73E+ 04	2.95E+ 03
November (*n* = 8)	2.60E+ 06	5.45E+ 03	6.39E+ 04	1.91E+ 04
Arithmetic mean (Overall)	1.63E+ 06	2.39E+ 03	2.87E+ 04	5.50E+ 03
Geometric mean (Overall)	1.12E+ 06	1.36E+ 03	1.66E+ 04	3.42E+ 03
***E. coli***				
May (*n* = 8)	2.19E+ 06	1.15E+ 03	2.04E+ 04	4.81E+ 03
June(*n* = 8)	1.80E+ 06	2.60E+ 02	2.73E+ 04	3.72E+ 03
July (*n* = 12)	4.38E+ 06	4.43E+ 02	4.56E+ 04	7.52E+ 03
August (*n* = 8)	3.62E+ 06	1.34E+ 03	4.26E+ 04	5.33E+ 03
September (*n* = 8)	4.84E+ 06	5.09E+ 03	1.05E+ 05	2.22E+ 04
October (*n* = 8)	4.84E+ 06	2.80E+ 03	1.34E+ 05	7.22E+ 03
November (*n* = 8)	4.84E+ 06	5.29E+ 03	2.46E+ 06	4.20E+ 04
^a^Arithmetic mean (Overall)	3.83E+ 06	2.21E+ 03	3.81E+ 05	1.29E+ 04
Geometric mean (Overall)	3.39E+ 06	1.15E+ 03	5.75E+ 04	7.46E+ 03

^a^ This mean was used to compute the risk of infection; n = number of samples collected/month, i.e. a single 500 ml sample was collected every 2 weeks from 4 sample sites to yield 8 samples per month except in July where samples were taken thrice to yield 12 samples. NB: the same samples were used to isolate *E.coli*/*Enterococcus*, total number of samples used was 60

The mean monthly *E. coli* concentration ranged from 2.60E+ 02/100 mL (in the effluent) to 4.84E+ 06/100 mL (in the influent). For enterococci, mean monthly MPN ranged from 2.60E+ 02/100 mL (in the effluent) to 3.19E+ 06/100 mL (in the influent) over the 7 months. Consideration of both arithmetic and geometric averages by sampling location revealed that the concentration of enterococci was highest at the influent followed by the upstream site, downstream site and was lowest at the effluent site (Table 1). Notably, some results associated with the *E. coli* influent samples were beyond the limit of detection of the method used. In such instances, the averages were calculated using the limit of detection per 100 mL.

For both organisms, the overall mean influent counts were significantly higher than the mean effluent counts (*p* ≤ 0.05). Similarly, the overall mean upstream counts were significantly higher than the mean downstream count (*p* ≤ 0.05).

### Molecular confirmation and characterization of *E. coli* and *Enterococcus* spp.

**Fig. 1:**
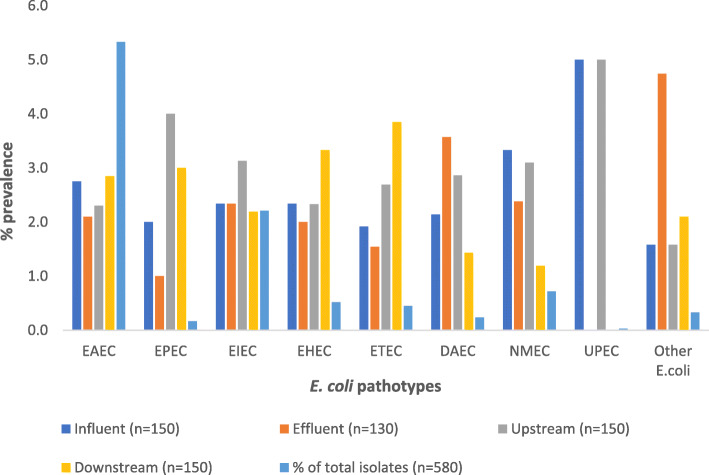
Prevalence of *E. coli* pathotypes at different sample sites

In total, 580 *E. coli* isolates from the effluent (*n* = 130), influent (*n* = 150), downstream (n = 150), and upstream (n = 150) sites were confirmed (*uid*A gene) using real-time PCR. The isolates were then delineated into different pathotypes using virulence genes. The percentage prevalence of the different pathotypes is shown in Fig. 1.

All the assayed virulence genes were detected except for the *stx* gene of Shiga toxin-producing *E. coli* (STEC). The *eagg* (309/580; 53.3%) and *Ipa*H (128/580; 22.1%), representing the EAEC and EIEC pathotypes, were the most prevalent genes from all sampled sites.

**Fig. 2:**
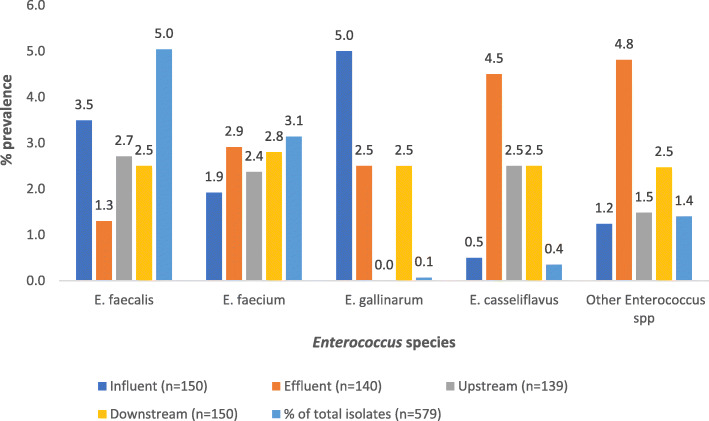
Prevalence of *Enterococcus* spp. at different sample sites

Also, 579 *Enterococcus* isolates were confirmed (*tuf* gene) using real-time PCR. The isolates were then delineated into different species using species-specific primers. The prevalence of the different species is shown in Fig. 2. *E. faecalis* (50.4%) and *E. faecium* (31.4%) were the most prevalent species.

### Probability of infection with *E. coli* following exposure to polluted river water and wastewater

**Fig. 3:**
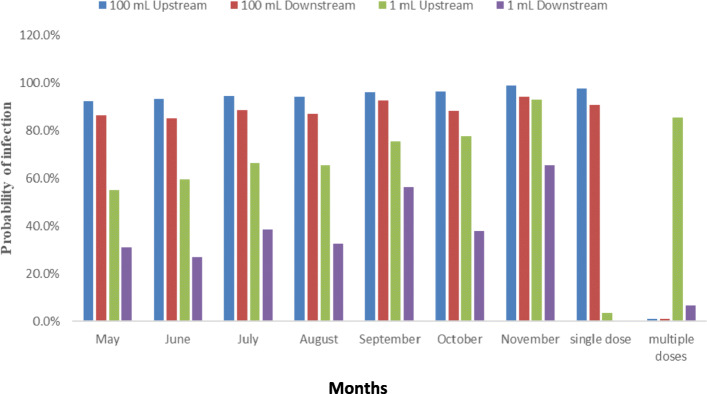
Probability of infection (Pi) with mean *E. coli* counts upstream and downstream of the WWTP based on single ingestion of I mL or 100 mL of river water (Also shown is the daily Pi based on single exposure and yearly Pi based on multiple weekly exposures)

The predicted Pi assuming both exposure scenarios is shown in Fig. 3.

The daily Pi with pathogenic *E. coli* following intentional uptake of 100 mL of the river water upstream and downstream from the investigated WWTP was found to be 97.6 and 90.8%, respectively. The monthly Pi was calculated for the upstream and downstream sites assuming once-off accidental ingestion of 1 mL or intentional ingestion of 100 mL based on monthly average *E. coli* counts obtained over the 7 months (Fig. 3). When assuming a 1 mL exposure volume, the monthly Pi for upstream and downstream sites ranged from 55.1 to 92.9% and 26.8 to 65.3%, respectively.

**Fig. 4:**
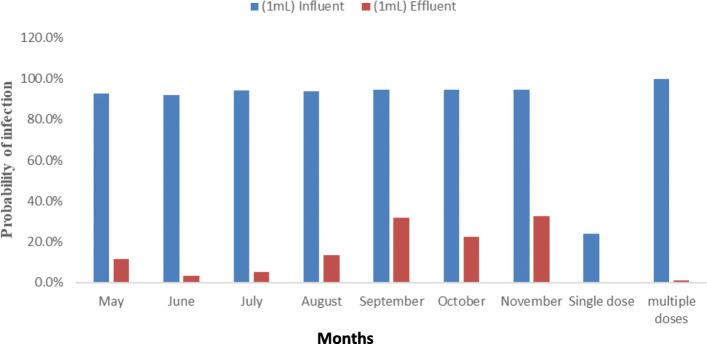
Probability of infection with mean *E. coli* counts based on accidental ingestion of 1 mL raw influent or 1 mL final effluent water in a WWTP (Also shown is the daily Pi based on single exposure and yearly Pi based on multiple weekly exposures)

The Pi was also calculated for the workers at the WWTP who were exposed to raw influent or final effluent based only on incidental ingestion or inhalation of a 1 mL contact volume (Fig. 4).

The projected daily and annual Pi with pathogenic *E. coli* for workers at the WWTP ranged from 0 to 1.2% for ingestion of effluent water and from 23.8–100% for accidental ingestion of raw influent water. The monthly Pi of workers at the WWTP based on accidental ingestion of raw influent or final effluent water ranged from 91.9–94.5% and 3.1–32.6% (Fig. 4). There was a significant difference between the Pi calculated for the upstream site and downstream site and the influent site and effluent site (*p* < 0.05). No statistically significant differences were observed in the Pi between adjacent months at the upstream and downstream sites, after exposure to 1 mL of polluted water (*p* > 0.05) or deliberate consumption of 100 mL of the water (p > 0.05).

## Discussion

Exposure to microbial pathogens in polluted waters has severe public health consequences. The current study evaluated the possible health hazards from the use of polluted river water by communities (mostly informal settlements) living upstream and downstream of a major WWTP, using *E. coli* as a model organism. The study also evaluated the risk of infection in WWTP workers, due to probable exposure to polluted aerosols. The mean microbial count at all the sampling sites exceeded the South African and WHO recommended limits for drinking and recreational water. Quantitative microbial risk assessment revealed that the waters upstream and downstream from the WWTP presented a high risk of infection for surrounding populations using these waters. The WWTP workers were also at risk of developing an infection from exposure to aerosols within the plant if they did not observe the strict use of personal protective equipment.

### Mean concentrations of *E. coli* and *Enterococcus* spp.

The mean *E. coli* and *Enterococcus* concentrations (Table 1) were above the established South African limits of 1000 CFU/100 mL and 0 CFU/100 mL for discharged wastewater and potable water for household use, respectively [24]. Based on the same guidelines, the concentrations of both organisms also exceeded the allowable limits for use in leisure and farming practices in South Africa. The counts recorded at the river sites also exceeded international standards. For example, the US Environmental Protection Agency (US EPA) recommends that the geometric mean of *E. coli* and *Enterococcus* in freshwater meant for recreational purposes should not exceed 126 CFU/100 mL and 35 CFU/100 mL, respectively [25]. Similarly, the WHO recommends that *E. coli* should not be detected in any water (100 mL) meant for human consumption [26]. This means that the river water upstream and downstream of the WWTP should be classified as generally unsafe for bathing and drinking purposes.

The area further up from the upstream sampling site is characterized by many industrial activities. This has led to the development of numerous informal settlements by the people working in the factories, and these settlements are located on one of the main tributaries of the Msunduzi River. Without adequate waste management facilities, these informal settlements dispose of their household and personal waste directly into the surface water body, resulting in high faecal pollution. Apart from the informal settlements, this area is also characterized by some agricultural activities around the river. The direct discharge of waste into the river and surface runoff from these agricultural activities could have contributed, as nonpoint sources of pollution, to the statistically significantly higher microbial counts recorded at the upstream site compared to the downstream site. Informal settlements [10] and agricultural activities [27] have been shown to negatively impact the quality of surface water bodies. Also, the lower microbial count at the downstream site compared to the upstream site could have resulted from the dilution effect from the large volume of treated wastewater discharged from the WWTP.

### Molecular characterization of *E. coli* and *Enterococcus* spp.

The continuous monitoring of wastewater treatment plants and their effectiveness in removing microbial pathogens remains an area of significant interest. *E. coli* remains the most widely used indicator of recent faecal contamination in aquatic environments and *i*s often used to monitor WWTPs effluents [5]. Despite the role played by WWTPs in treating wastewater before discharge into receiving surface waters, the importance of WWTPs in the dissemination of pathogens into surface water has been reported in previous studies [8, 9]. In the current study, seven groups of pathogenic *E. coli* (InPEC: EAEC, EPEC, EIEC, EHEC (O157H7), ETEC, DAEC; and ExPEC: NMEC and UPEC) were isolated from surface waters (upstream and downstream) as well as at the influent and final effluent of the studied WWTP. The prevalence of the pathogenic *E. coli* strains varied across the different sampling points (Fig. 1). Interestingly all pathogenic *E. coli* strains were detected at all sampling points, including surface waters upstream and downstream of the wastewater treatment plant. This points to the contamination of the Msunduzi River with pathogenic *E. coli* strains upstream and downstream of the studied WWTP, presenting a significant public health risk, especially for communities living along the riverbanks. These findings also imply that *E. coli* strains which are probably released from the wastewater treatment process could survive in the environment.

In the current study, InPEC were the predominant isolates detected across all sampled points with a frequency of detection ranging from 1.7–53.3%. The InPEC isolates accounted for 89.1% of all the isolates with ExPEC accounting for 7.6% and the uncharacterized isolates 3.3%. These findings are contrary to the findings of an Australian study in which no InPEC isolates were detected in different treatment tanks of four STPs [28]. The same study reported a higher prevalence of ExPEC isolates (59.5%), specifically UPEC isolates across the four STPs. InPEC isolates have, however, been shown to survive the wastewater treatment process. A study done in Gauteng province on six WWTPs tested water samples, for EPEC, EIEC, EAEC, EHEC and ETEC, from raw sewage, primary, secondary, and tertiary effluents. InPEC isolates were detected in all 6 WWTPs [13]. All five pathotypes were detected at various stages of the treatment process, including in the tertiary effluent, showing that InPEC isolates do survive the wastewater treatment process. Another study done in South Africa focused on pathogenic *E. coli* from the final effluents of two WWTPs in the Eastern Cape province and reported a higher proportion of ExPEC isolates (56.5%) than InPEC (16,6%) isolates [9]. In a study by Frigon et al. (2013), the reported proportions of InPEC and ExPEC were 10 and 24% respectively. The differences in the proportions of InPEC to ExPEC between the current study and the other studies could be due to the number of medical facilities in the uMgungundlovu District, most of whose effluent is treated at the WWTP we investigated. The district has nine major hospitals, 46 fixed clinics, and three community health centres.

The detection of *eaeA + fliCH7* genes, which are characteristics of EHEC isolates, specifically O157:H7, at all sampled sites is a cause for concern (Fig. 1)*.* The EHEC group has been recognized as the universal cause of serious human gastrointestinal diseases [29]. EHEC O157:H7 /H(−) (nonmotile) can occur as variants that either possess the Shiga toxin gene (*stx*) or lack it in clinical or environmental isolates [30]. The *stx* (*stx*1 and *stx*2) genes [31] together with the intimin protein (*eae*A gene), which is involved in bacterial adherence, are some of the virulence determinants that can be used to identify EHEC strains. The *fliCH7* gene encodes the structural flagella antigen H7 in *E. coli* and is used to identify the OH157: H7 serotype [32]. The *stx*, *eaeA* and *fliCH7* genes were used in this study for the molecular identification of OH157: H7. All the detected OH157: H7 strains were, however, *stx* negative. Reports on the isolation of O157:H7 are rare in South Africa, particularly from environmental samples [33, 34] . Muller et al. [35] used immunomagnetic separation (IMS) in combination with selective media and other techniques to evaluate the incidence of *E. coli* O157:H7 in sewage and environmental water samples in Gauteng South Africa. The authors only found the *E. coli* O157:H7 in 1.1% of the 16 sewage samples and did not find any in the 40 river water samples analyzed. In an earlier study, Müller et al. [36] assessed the presence of *E. coli* O157:H7 in river water used for domestic purposes in the same province. Out of the 204 water samples assayed, none of the 633 suspected *E. coli* O157:H7 had all the requisite virulence determinants necessary for *E. coli* O157:H7 classification. In another study on *E. coli* O157:H7 that included an investigation of 180 drinking water samples from the Amathole District in South Africa, only 4 (2.2%) were confirmed to be positive using molecular techniques [33]. The detection of *E. coli* O157:H7 in all the sampled sites in the present study, albeit at very low prevalence (5.2%), is consistent with the generally low occurrence of the organisms in environmental samples in South Africa [34].

The EAEC were the most prevalent DEC pathotype across all sampled points (53.3%; 309/580 isolates), followed by EIEC (22.1%; 128 isolates), EHEC (5.2%; 30 isolates), ETEC (4.5%; 26 isolates), DAEC (2.4%; 14), and atypical EPEC/EHEC (1.7%; 10). The proportions of DEC seem to vary across different studies. Osińska et al. [37] characterized *E. coli* obtained from raw and processed wastewater from a WWTP and its receiving waters in Poland using antibiotic resistance and virulence genes. From the 317 *E. coli* isolates assayed in their study, they found the following prevalences: typical EPEC bfpA (65%), *eae*A (39%); ETEC, *st* (56%); EHEC, *stx* (9%); EIEC, *ipa*H (5%); EAEC, CVD432 (28%). The observed prevalence of the different pathotypes was different from that observed in the current study. The differences could be due to the difference in the target genes used, the number of isolates assayed and the geographical location. Omar and Barnard [13] developed an 11-gene-single-step multiplex PCR for the detection of DEC in clinical and environmental water samples. In their study they examined 291 wastewater samples for DEC and found the following prevalences: EPEC/EHEC (*eaeA* gene) 40%, EAEC (*eagg*) 35%, EHEC (*stx*1/*stx*2) 12.4%, ETEC (*lt*/*st*) 11.3% and EIEC (*ial*) 2%.. The study by Omar and Barnard [13] did not assay for DAEC. Another study done in Tunisia analyzed 60 wastewater samples from 15 WWTPs targeting the influent and final effluent [38]. The authors found that from the 30 influent samples, ETEC isolates were the most prevalent (53.3%), followed by EAEC (16.6%), and EIEC (6.6%). At the effluent points, the ETEC (53.3%) was still the most isolated pathotype followed by EAEC (33.3%) and EIEC (13.3%). The EHEC was not detected in any of the sampled points. In the study by Adesifoye and Okoh [9] in South Africa, the following proportions of DEC were observed in the final effluent of two WWTPs from a total of 223 *E. coli* isolates: atypical EPEC/EHEC (7.6%), ETEC (1.4%), and EAEC (7.6%). The EIEC and DAEC were not detected at all. Our study is one of the few from this region to report on the presence of DAEC in environmental water samples. The prevalence of DEC in environmental water samples possibly varies from region to region and is probably dependent on the communities served by the WWTPs, among other factors.

Characterization of the enterococci, according to species, revealed the presence of four species at the assayed sites. *E. faecalis* (50.4%) was the dominant species across all sampled points, followed by *E. faecium* (31.4%) which was, however, the most abundant from effluent samples (Fig. 2). Our findings are similar to those of a study done in Ireland by Cheng et al. [39], which examined the seasonal incidence of *E. faecalis* and *E. faecium* in raw and treated effluent as well as from biosolids in four WWTPs. They observed a higher prevalence of *E. faecalis* at all sampled sites when both species were detected. Similar findings were reported by Alipour et al. [40] in a study done in Iran on surface water (river and coastal) in which they found *E. faecalis* to be the predominant species (68.6%) out of a total of 70 *Enterococcus* isolates. The other species reported in that study were *E. faecium* (20%), *E. gallinarum* (7.1%), and *E. casseliflavus* (4.3%). This varies with findings from our study as *E. casseliflavus* (3.5%) was more prevalent than *E. gallinarum* (0.7%) (Fig. 2). Iweriebor et al. [41] conducted a study on enterococci from hospital effluent as well as from the final effluent of a WWTP in Eastern Cape, South Africa. From a total of 62 confirmed *Enterococcus* isolates, they found *E. faecalis* (48.39%) as the dominant species followed by *E. durans* (24%) but did not identify other species, a finding which differed from our study.

The discrepancies between the current study and other studies could be due to several factors like the number of samples analyzed, the number of isolates characterized, the isolation and characterization methods used, the frequency of sampling, seasonal differences during sampling, wastewater treatment processes used, and other physicochemical parameters of the water sampled, among others.

### Probability of infection with *E. coli* following exposure to polluted river water and wastewater

A total of 580 *E. coli* isolates were analyzed for their pathogenic potentials, using specific virulence genes. Although this number was large, it was not representative of the total number of *E. coli* isolated in the entire study. Therefore, to calculate the risk of infection in this study, it was assumed that 8% of the mean *E. coli* concentration was pathogenic, based on literature [10, 22]. The hazards considered for the QMRA were the six diarrhoeagenic *E. coli* (DEC) pathotypes for which the incidence and probability of infection due to contact with wastewater were estimated. All these pathotypes are characterized by the fecal-oral transmission route. The DEC pathotypes were chosen for QMRA based on their reported low infectious doses, ease of detection, and quantification [2, 22]. The pathogens can also persist for weeks in the environment and are difficult to inactivate or remove adequately using conventional wastewater treatment processes [8, 9, 37].

For the upstream and downstream sites based on the unintentional consumption of 1 mL or the deliberate consumption of 100 mL of river water, the results showed that the annual Pi with pathogenic *E. coli* was above that recommended for potable water [10, 4] by the WHO [42]. The Pi was significantly higher in the upstream site than downstream site assuming exposure to either a 1 mL or 100 mL volume, implying that communities upstream of the WWTP were at greater risk of infection. Assuming exposure to 1 mL every week for a year (52 weeks), the probability of infection at the downstream site was found to be 6.6% compared to 85.5% at the upstream site (Fig. 3). These results are comparable to work done in Gauteng, South Africa where the health hazards in four rivers and their associated WWTPs, utilizing waterborne *Salmonella enterica* serovar Typhimurium, *Shigella dysenteriae,* and *Vibrio cholerae*, was determined [43]. The authors reported that the day-to-day and yearly joint risk of infection resulting from the consumption of 1 mL of raw water was higher at the upstream than downstream sites [43]. The study also described the hazard resulting from drinking 50 mL and 100 mL as having an annual joint pathogen infection risk of 100%. The current study also recorded an annual risk of infection of 100% following exposure to 100 mL of polluted untreated river water, for both the upstream and downstream sites (Fig. 3). In another study done in South Africa at sites along the Apies river, the risk of infection with *E. coli* ranged from 6 to 84% across the sampled sites, assuming a one-off intake of 100 mL; the risk increased to 100% at eight of the ten sites assuming multiple exposures (weekly) over 6 months [10]. These findings are like those in the current study regarding the Pi following multiple exposures and assuming a 100 mL ingestion volume (Fig. 3). However, the risk of infection was calculated over a more extended period (52 weeks) in the present study. Assuming a single dose of 100 mL, the monthly Pi ranged from 92.3 to 98.8% for the upstream site and 85 to 94.2% for the downstream site. This is higher than the Pi stated by Abia et al. [10] mainly because they sampled from more sites, some of which were not near WWTPs and had a lower bacterial load. The findings of the current study also differ and are higher than those reported by two studies done in the Olifants River, South Africa [44, 45]. Genthe et al. [44] stated that the Pi with *E. coli* fluctuated from 8 to 80% across the 12 studied sites while le Roux et al. [46] described a combined Pi of between 1 and 26% for the seven pathogens investigated due to the drinking of 100 mL of unprocessed water from the upper Olifants River. Based on *E. coli* as the surrogate for pathogens, the Pi in the study of le Roux et al. [46], at some locations was approximately 80% assuming a once-off exposure incident. This is lower than the Pi in the current study, which stood at 90.8 and 97.6% for the downstream and upstream sites, respectively (Fig. 3).

Wastewater treatment plants are considered a potential hazard for human health, particularly for WWTP workers [47]. The hazards are considered to be dependent on the characteristics of the wastewater, method of treatment, type of apparatus used, as well as the meteorological and climatic conditions [46, 47]. WWTP workers may be exposed to biohazards through bioaerosols, aeroallergens, and other airborne organic particles which can potentially cause gastroenteritis and pulmonary diseases [17, 47]. Despite the identification of health hazards connected to exposure to aerosols, risk assessment is still challenging as the degree of exposure to bioaerosols in WWTP varies depending on various factors [48]. There is a paucity of data regarding the risk of exposure plant workers face in WWTPs, especially from developing countries [48]. Thus, the current study also estimated the occupational risk of infection with pathogenic *E. coli* for workers that may be exposed to wastewater in the WWTP. The risk assessment was surmised on the accidental ingestion of 1 mL of either influent or effluent water during normal work activities. The Pi resulting from a once-off unintentional consumption of 1 mL of effluent was 0, and 23.8% for raw influent water (Fig. 4). Multiple weekly exposures of the same volume over a year resulted in an estimated Pi of 1.2 and 100% for the effluent and influent, respectively. The low Pi observed for the effluent is due to the significantly lower microbial load recorded in the final effluent throughout the study (Table 1), pointing to the efficiency of the WWTP in reducing the microbial load. The monthly Pi was calculated assuming a single exposure event, i.e. accidental consumption of 1 mL of water; generally, the estimated risk was significantly higher for influent water (Fig. 4). Bioaerosol emissions have been found to be the highest in pre-treatment and aerated grit separation stages and to decrease in subsequent treatment stages [49]. This implies WWTP workers are more likely to be exposed to bioaerosols from raw influent water than effluent water, cementing the need for strict adherence and proper use of personal protective equipment. Other exposure control measures in WWTPs could include preventing workers from spending long periods in areas with high bioaerosol emissions [17].

It should be noted, however, that the 8% pathogenic potential assumed in the current study may have underestimated or overestimated the risk of infection [6]. Also, the parameters used in the current study were for *E. coli* O157:H7. Therefore, the risk of infection estimated in this study should not be generalized for all other pathogenic microbes that are present in wastewater and polluted river waters. It was also assumed that every exposed individual would have the same chance of getting sick. This is not always the case as other factors like the age, the immune status, infective dose of the specific pathogen (among others) could influence whether an individual develops an infection following exposure to a pathogen. Thus, we recommend that future studies look at using other pathogens.

## Conclusion

The findings of the present study show that the investigated WWTP and its associated surface water had a high percentage of pathogenic *E. coli*. The river water upstream and downstream of the investigated WWTP is contaminated with high concentrations of fecal indicator bacteria, and there is a substantial risk of infection with pathogenic *E. coli* for people that use these water sources. An occupational health hazard was noted for workers at the WWTP, especially if they were exposed to raw influent wastewater. The study brings to light the need for relevant authorities to work towards ensuring that all communities are supplied with clean water for domestic and recreational purposes, and seeks to encourage the development of mitigation strategies in resource-limited communities. The study further highlights the need to institute occupational health and safety measures for WWTP workers.

## Methods

### Study site and sample collection

The WWTP serves the uMsunduzi municipality and has a biological treatment capability of 65 Mℓ/day. The WWTP is located along the uMsunduzi river and uses aeration basins for organic nutrient elimination and clarifiers for the separation process. Clarified effluent undergoes tertiary treatment, which involves chlorination [50]. The uMsunduzi River passes through Pietermaritzburg the provincial capital of KwaZulu-Natal. It receives runoff from the municipality, rural communities, agricultural areas, and treated effluent from the investigated WWTP [15]. The river is also one of the main tributaries of the Umgeni River, both rivers are key sources of water that is used for recreational, domestic, agricultural, and industrial purposes in KwaZulu-Natal [15]. The climate in KwaZulu-Natal is typically tropical to sub-tropical with rainfall falling throughout the year, with the least rainfall experienced between May and August. Water sampling was done fortnightly over 7 months (May 2018 to November 2018). A total of 60 grab water samples were collected in sterile 500-mL containers, from the influent (29°36′3.70″S 30°25′41.71″E) and final effluent (29°35′49.97″S 30°26′19.74″E) of a major WWTP and the Msunduzi River, upstream (29°36′10.73″S 30°25′29.97″E), and downstream (29°36′27.54″S 30°27′0.76″E) from the WWTP in uMgungundlovu District, in KwaZulu-Natal Province, South Africa. Fifteen samples were collected from each sampling site. Water samples were transported to the research laboratory at 4 °C for subsequent microbial analysis.

### Fecal indicator analysis

Detection and quantification of *E. coli* and *Enterococcus* were performed using the Colilert®-18 / Quanti-Tray® 2000 system and Enterolert® / Quanti-Tray® 2000 systems, respectively according to the manufacturer’s instructions (IDEXX Laboratories (Pty) Ltd., Johannesburg, South Africa). Positive results for both organisms were denoted by fluorescent blue wells under UV light at 300 nm. Positive wells were then used to determine the most probable number (MPN) of each organism in 100 mL of water sample (MPN/100 mL). Both Quanti-Tray methods had a limit of detection equivalent to 4.84E+ 06/100 ml.

### Isolation of *E. coli* and *Enterococcus* spp.

Positive well contents were sub-cultured onto selective media as previously described [51] and. *E. coli* was sub-cultured on Eosin Methylene Blue (EMB) and incubated at 37 °C for 18 - 24 h while *Enterococcus* was sub-cultured on Bile Aesculin Azide Agar (Merck, Germany) or Slanetz and Bartley agar (Merck, Germany) and incubated at 41 °C for 24 h – 48 h. At least ten distinct colonies representing each sampling site were randomly selected from each media type and further sub-cultured onto respective selective media to obtain pure colonies. Presumptive isolates were stored in 20% glycerol stocks at − 80 °C for further investigation.

### DNA extraction and molecular confirmation

Bacterial DNA for both *E. coli* and *Enterococcus* spp. was extracted using a standard heat-lysis protocol. Molecular confirmation of all isolates was done using real-time PCR, where the *uid*A (β-D glucuronidase) gene was used for *E. coli* [52] and the *tuf* gene for enterococci [53]. *E. coli* (ATCC® 25922) and *E. faecalis* (ATCC 51299) served as positive controls.

### Determination of *E. coli* pathotypes

Delineation of *E. coli* into various pathotypes was done using real-time PCR. The pathotypes assayed for included EPEC, ETEC, EAEC, EIEC, DAEC, EHEC, NMEC, and UPEC. Conventional PCR was used to assay the DAEC and UPEC pathotypes. All reactions included a no-template control consisting of the reaction mixture. The primers for virulence genes and reference strains used in this study are shown in Table S1 (Supplementary materials). All real-time PCR assays consisted of 5 μL 2x Luna universal master mix (New England Biolabs, South Africa), 0.5 μL (1.25 μM) of each primer, 3 μL of template DNA which was topped up to 10 μL with nuclease-free water except for the *St* gene where 1 μL (2.5 μM) of each primer and 2 μL of genomic DNA was used. The following real-time PCR settings were used: initial hold at 50 °C for 2 min; 35 cycles {denaturation at 95 °C for 2 min; annealing at 60 °C for 15 s; extension at 72 °C for 10 s}; final extension at 72 °C for 5 min. All real-time PCR assays were done on an Applied Biosystems QuantStudio 5 Real-time PCR system (Thermo Fisher Scientific, Waltman, Massachusetts, USA).

Conventional PCR was done in a 20 μL reaction volume with 2 μL of genomic DNA, 10 μL of One Taq master mix (New England Biolabs, South Africa), 0.5 μL (0.6 μM) of each primer, plus 7 μL of nuclease-free water. The PCR reactions were as follows: initial denaturation (94 °C, 30s), 35 cycles of denaturation (94 °C, 15 s), annealing (59 °C, 30s) for the *daaE* gene (DAEC), (55 °C, 30s) for the *papC* gene (UPEC), extension (68 °C, 45 s), final extension (68 °C, 5 min). PCR products were run on a 1% ethidium bromide-stained gel with a 100 bp ladder (Thermo Fisher Scientific, USA) in TAE buffer for 1 h at 100 V and visualized using a Gel Doc™XR+ imaging system (Bio-Rad, South Africa).

### Speciation of *Enterococcus* isolates

*Enterococcus* isolates were further characterized into various species using real-time PCR, according to [54] (Table S2, Supplementary materials). All reactions included a no template control. All PCR assays comprised 5 μL of Luna universal master mix 0.5 μL (1.25 μM) of the individual primer, 3 μL of template DNA, and topped up to 10 μL with nuclease-free water. The PCR conditions were initial hold at 50 °C (2 min); 35 cycles {denaturation at 95 °C (2 min); annealing at 60 °C (15 s); extension at 72 °C (10 s)}; final extension at 72 °C for 5 min. A melt curve was prepared, according to Molechan et al. [55]. All qPCR reactions were done on the Applied Biosystems QuantStudio 5 Real-time PCR system.

### Risk assessment

The health hazard related to exposure to waterborne *E. coli* at the WWTP and its associated surface water was computed using a beta-Poisson response model for *E. coli* O157:H7. The probability of infection (Pi) was based on the unintentional or deliberate consumption of predetermined quantities of river water associated with the studied WWTP. The Pi with pathogenic *E. coli* resulting from a single unintentional consumption of 1 mL and deliberate consumption of 100 mL of water from the Msunduzi River (upstream and downstream of the WWTP) was calculated for the communities which rely on the river for their domestic and recreational needs. The calculations were based on a once-off exposure (daily risk) and repeated exposure (weekly exposure) over 12 months. Pi was also calculated based only on the unintentional ingestion (through inhalation) of 1 mL of water by the workers at the WWTP [16]. The Pi was calculated based on the postulation that only 8% of the total *E. coli* population was pathogenic [10, 16, 22]. The formulae used to calculate the Pi following the once-off consumption and Pi ensuing from numerous exposures are presented in Table 2. Manifold exposures were computed weekly (*n* = 52) over 12 months. The pathogen infectivity constants β and α are presented in Table 2.

**Table 2:** Beta-Poisson response model and parameters used for quantitative microbial risk assessment

Microorganism	Parameters	Dose-response model	References
*Escherichia coli*	β = 2.473, α = 0.395	P (inf) = 1 – (1 + N/β)-α	[16, 56]
	*n* = 52	P(n) = 1 – (1 – Pinf) n	[16]

Key: *P (inf)* Probability of infection; *P(n)* annual probability of infection; *N* dosage (number of microbes ingested); *n* sum of exposures that occurred

### Statistical analysis

All statistical analysis was done using Microsoft Excel 2018 and Statistical Package for Social Sciences 23 (SPSS, IBM Corporation, Armonk, New York, USA). A paired t-test was used to ascertain for any significant differences in the mean microbial counts and the Pi between the sites – upstream and downstream, and influent and effluent. A paired t-test was also done to ascertain any significant differences in the Pi between adjacent months in the event of accidental ingestion of 1 mL or intentional ingestion of 100 mL. All statistics were considered significant at *p* ≤ 0.05.

## Supplementary Information

**Additional file 1 **: **Table S1:** *E. coli* primers and reference strains used in PCR reactions. **Table S2:** *Enterococcus* primers and reference strains used in PCR reactions**. (DOCX 18 kb)**

## Data Availability

The data generated and analyzed in this study are available upon request.

## Abbreviations

PCR: Polymerase chain reaction
CFU: Colony forming unit
MPN: Most probable number
WWTP: Wastewater treatment plant
qPCR: Quantitative polymerase chain reaction
QMRA: Quantitative microbial risk assessment
ExPEC: Extraintestinal pathogenic *E. coli*
UPEC: Uropathogenic *E. coli*
APEC: Avian pathogenic *E. coli*
NMEC: Neonatal meningitis *E. coli*
InPEC: Intestinal pathogenic *E. coli*
DEC: Diarrhoeagenic *E. coli*
EHEC: Entero-hemorrhagic *E. coli*
EPEC: Entero-pathogenic *E. coli*
EAEC: Entero-aggregative *E. coli*
ETEC: Enterotoxigenic *E. coli*
EIEC: Entero-invasive *E. coli*
DAEC: Diffusely adherent *E. coli*
