# Supplementary material for: Quantitative microbial risk assessment for waterborne pathogens in a wastewater treatment plant and its receiving surface water body
Source: BMC Microbiol. 2020 Nov 12;20:346. doi: 10.1186/s12866-020-02036-7 (PMC7663859; doi:10.1186/s12866-020-02036-7)
Supplement: Supplementary file 1 — Additional file 1 : Table S1: E. coli primers and reference strains used in PCR reactions. Table S2: Enterococcus primers and reference strains used in PCR reactions. (DOCX 18 kb) [file 12866_2020_2036_MOESM1_ESM.docx]

**Table S1:** *E. coli* Primers and reference strains used in PCR reactions

| **Pathogen** | **Name of gene** | **Primer sequence (5’- 3’)** | **Reference strain** | **References** |
| --- | --- | --- | --- | --- |
| EPEC/EHEC | *eaeA* | (F) ATGCTTAGTGCTGGTTTAGG | DSM8695 | Stanilova et al. (2011) |
|  |  | (R) GCCTTCATCATTTCGCTTTC |  |  |
| EAEC | *éagg* | (F) AGACTCTGGCGAAAGACTGTATC | DSM10974 | Pass and Odedra (2004) |
|  |  | (R) ATGGCTGTCTAATAGATGAGAAC |  |  |
| EIEC | *ípaH* | (F) GTTCCTTGACCGCCTTTCCGATACCGTC | DSM9025 | Vidal et al. (2005) |
|  |  | (R) GCCGGTCAGCCACCCTCTGAGAGTAC |  |  |
| EHEC | *fliCH7* | (F) TACCATCGCAAAAGCAACTCC | O157H7 | Cebula et al. (1995) |
|  |  | (R) GTCGGCAACGTTAGTGATACC |  |  |
|  | *sxt* | (F) GAGCGAAATAATTTATATGTG |  |  |
|  |  | (R) TGATGATGGCAATTCAGTAT |  |  |
| ETEC | *St* | (F) TTTCCCCTCTTTTAGTCAGTCAACTG | DSM10973 | Stacy-Phipps et al. (1995) |
|  |  | (R) GGCAGGATTACAACAAAGTTCACA |  |  |
|  | *Lt* | (F) TGCTATGTGCATACGGAGC |  |  |
|  |  | (R) CCATACTGATTGCCGCAAT |  |  |
| DAEC | *daaE* | (F) GAACGTTGGTTAATGTGGGGTAA | - | Vidal et al. (2005) |
|  |  | (R) TATTCACCGGTCGGTTATCAGT |  |  |
| NMEC | *IbeA* | (F) TGGAACCCCGCTCGTAATATAC | DSM10819 | Cebula et al. (1995) |
|  |  | (R) CTGCCTGTTCAAGCATTGCA |  |  |
| UPEC | *papC* | (F) GACGGCTGTACTGCAGGGTGGCG | DSM4816 | Hilali et al. (2002) |
|  |  | (R) ATATCCTTTCTGCAGGGATGCAATA |  |  |

**Key**: F – Forward primer; R – Reverse primer

**Table S2:** *Enterococcus* primers and reference strains used in PCR reactions

| **Strain** | **Primer** | **Sequence (5’-3’)** | **Reference strain** | **References** |
| --- | --- | --- | --- | --- |
| *E. faecalis* | FL1  FL2 | ACTTATGTGACTAACTTAACC  TAATGGTGAATCTTGGTTTGG | *E. faecalis* ATCC 51299 | Jackson et al. (2004) |
| *E. faecium* | FM1  FM2 | GAAAAAACAATAGAAGAATTAT  TGCTTTTTTGAATTCTTCTTTA | *E. faecium* ATCC 35667 | Jackson et al. (2004) |
| *E. gallinarum* | GA1  GA2 | TTACTTGCTGATTTTGATTCG TGAATTCTTCTTTGAAATCAG | *E. gallinarum* (NHLS, SA field strain) | Jackson et al. (2004) |
| *E. casseliflavus* | CA1  CA2 | TCCTGAATTAGGTGAAAAAAC  GCTAGTTTACCGTCTTTAACG | *E. casseliflavus* ATCC 700327 | Jackson et al. (2004) |

**Key**: NHLS, SA - National Health Laboratory Services, South Africa
